# A comparative content analysis of media reporting of sports betting in Australia: lessons for public health media advocacy approaches

**DOI:** 10.1186/s12889-017-4866-7

**Published:** 2017-11-14

**Authors:** Jennifer L. David, Samantha L. Thomas, Melanie Randle, Steven J. Bowe, Mike Daube

**Affiliations:** 1 Centre for Population Health Research, School of Health and Social Development, Faculty of Health, Deakin University, Geelong, Australia; 2 School of Management, Operations and Marketing, Faculty of Business, University of Wollongong, Wollongong, Australia; 3 Biostatistics Unit, Faculty of Health, Deakin University, Geelong, Australia; 4 Faculty of Health Sciences, Curtin University, Perth, Australia

**Keywords:** Sports betting, Media analysis, Australia, Industry, Framing, Advocacy

## Abstract

**Background:**

Harmful gambling is a significant public health issue. There has been widespread discussion in the Australian media about the extent and impact of sports betting on the Australian community, particularly relating to young men and children. Given the role that the media plays in influencing policy change and political agendas, and the acknowledgement that media based advocacy is a fundamental component of successful advocacy campaigns, this research aimed to investigate how different stakeholder groups discuss sports betting within the Australian print media. The study uses this information to provide recommendations to guide public health media advocacy approaches.

**Methods:**

A quantitative content analysis of print media articles was conducted during two significant Parliamentary Inquiries about sports betting - (1) *The Joint Select Committee Inquiry into the Advertising and Promotion of Gambling Services in Sport* (2012/2013), and (2) *'The Review of Illegal Offshore Wagering* (2015/2016). A total of 241 articles from 12 daily Australian newspapers were analysed. Statistical analysis was used to compare frequency of, and changes in, themes, voices and perspectives over time.

**Results:**

Discussions about the marketing and communication of sports betting was a main theme in media reporting (*n* = 165, 68.5%), while discussions about gambling reform decreased significantly across the two time periods (*p* < 0.0001). The presence of sports betting industry (*p* < 0.0001), sporting code (*p* < 0.0001) and public health expert (*p* = 0.001) voices all increased significantly across the two time periods. There were very few (*n* = 11, 4.6%) voices from those who had experienced gambling harm. Finally, while there were significantly fewer articles taking the perspective that regulation changes were needed to protect vulnerable sub-populations (*p* < 0.0001), articles that had a neutral perspective about the need for regulation change increased significantly across the two time periods (*p* < 0.0001).

**Discussion and conclusions:**

Mapping the media reporting of sports betting is important in developing effective public health advocacy approaches. This study indicates that discussions about the marketing strategies utilised by the sports betting industry was still a main theme in media articles. However, discussions relating to sports betting reforms, in particular to protect individuals who may be vulnerable to the harm associated with these products and their promotional strategies (for example children and young men) decreased during the time periods. Public health advocates may seek to address the decrease in media reports about reform by developing clear evidence-based messages about why regulatory reform is needed, as well as the potential consequences of not implementing reforms. Working with organisations to build capacity for people who have experienced gambling harm may help ensure that individuals with a lived experience of harm have an increased voice in the media.

## Background

### Harmful gambling and public health

In recent years there has been increased recognition that the harms associated with commercial gambling go beyond individual pathology and may have significant negative impacts for families, communities and populations [1]. For example, recent research has concluded that the financial and social burdens associated with gambling harm in Australia are comparable to the financial and social burdens associated with alcohol misuse and depression [2]. While gambling related harm is generally defined as any adverse consequences associated with gambling [1], a team of international experts have defined ‘harmful gambling’ as:

*“…any type of repetitive gambling that an individual engages in that leads to* [or aggravates] *recurring negative consequences such as significant financial problems, addiction, as well as physical and mental health issues…*[and that may] *also be experienced by the gambler’s family, social network, and community”* [3, p. 5].

These newer conceptualisations of the negative impacts of gambling (and its promotion) across communities and populations [4–6], have also led to the development and endorsement of public health models that take into consideration the broad range of individual, socio-cultural, environmental, and commercial factors, which may stimulate harmful patterns of gambling [3, 7, 8]. For example, the Public Health Association of Australia (PHAA) recommends the adoption of a public health model for gambling that “emphasises the general protection and promotion of well-being in the community” [9, p. 5], while the Victorian Responsible Gambling Foundation (VRGF) highlights the need for a public health-based approach to the prevention of gambling related harm [10]. As with many other areas of public health that have sought to reduce the harms associated with commercialised products (such as tobacco, alcohol, and junk food), advocacy is likely to form an important part of any public health initiatives to respond to harmful gambling. This is because, as Chapman [2001] argues, in every branch of public health, advocacy has played a critical role in the translation of research into policy and practice, and in stimulating community support for regulatory reform [11]. However, there is limited discussion in the public health literature to guide a range of advocacy initiatives seeking to respond to harmful gambling.

### The changing Australian gambling environment

Over $22 billion was lost on gambling in Australia in 2014/15 [12]. Approximately 80,000 to 160,000 Australian adults experience problems with gambling each year, with another 230,000 to 350,000 people at significant risk of developing a problem with gambling [13]. Further, for each individual that develops a problem with gambling, it is estimated that up to ten others in their social network will be negatively impacted by gambling harm [13]. While these figures are still largely attributed to losses on Electronic Gambling Machines (EGMs, or poker machines), in recent years there has been increasing concern about the harms associated with newer, more pervasive forms of gambling such as online sports betting [14, 15]. There are approximately 25 corporate bookmakers in Australia, the majority of which are registered in the Northern Territory [16]. Recent data suggests that the losses from sports betting increased by 30% from $626 million 2012/13 to $815 million in 2014/15 [12]. While participation rates in other forms of gambling such as EGMs have declined, state-based prevalence studies indicate that participation in sports and event betting by problem gamblers significantly increased from 15.66% in 2008 to 45.41% in 2014, and that overall participation rates in young men increased from 6.53% in 2008 to 8.65% in 2014 [17].

Two key issues have attracted national government and media attention in Australia in relation to sports betting – the promotion of sports betting, and illegal offshore wagering.

### The impact of the promotion of sports betting in Australia

Discussion about the impact of the marketing of sports betting products has led to the most debate about gambling in Australia in recent years [14, 18–20]. In 2015, Standard Media Index data showed that $236 million was spent on gambling advertising, the majority of which was on betting products [21]. This figure does not take into account sponsorship deals between corporate bookmakers and sporting codes [22]. Researchers have argued that marketing strategies aligned with sports betting are particularly pervasive because in essence, sport is the product, with betting the gambling service linked to that product [18, 23]. In particular, research has demonstrated the significant impact of sports betting marketing on the gambling attitudes and consumption intentions of young men and children. Researchers investigating the content of advertising have found that young men aged 18–35 are the key target audience for betting companies [23], and that some young men felt that promotions encouraged them to gamble [6] and perceive that sports betting has become part of their sporting rituals [14].

Research has also explored children’s recall and awareness of sports betting marketing. Studies have shown that children are able to identify clearly gambling sponsorship aligned to Australia’s major sporting codes [24], to name multiple sports betting brands [25], and to recall specific promotions within advertisements such as inducements, and believe that gambling is a normal part of sport [18]. Research also indicates that parents believe the marketing of gambling during sport encourages young people to view sport through a ‘gambling lens’ [5, p. 8]. Researchers and politicians have increasingly called for the closure of a loophole that allows sports betting advertisements during televised sporting events or current affairs programs prior to the 8:30 pm watershed (the time after which adult content may be broadcast), although sports betting advertisements are banned during all other programs and timeslots considered for general exhibition and have a general classification (G) [26].

### Illegal and offshore wagering

There has been some focus on illegal and offshore betting on sports, with the gambling industry and sporting codes arguing for more relaxed regulations in Australia (such as allowing in-play betting) to prevent consumers betting on sports via illegal offshore bookmakers [27]. While some have cited figures of up to $1 billion gambled on offshore betting sites [28, 29], the Australian Government has noted that there is significant uncertainty associated with figures regarding offshore gambling markets, as “estimates are generally based on extrapolating from the available data based on the estimated number of operators, potential consumers, self-reported participation and other factors” [30, p. 32]. Further, statistics suggest that less than 5% of total gambling expenditure in Australia is via illegal or offshore betting sites, and that there is no evidence to suggest that gambling risk is increased in relation to the particular use of offshore betting sites [31].

### Parliamentary Inquiries into sports betting and offshore and illegal wagering

In line with increasing concern about the promotion of sports betting and illegal offshore gambling, two Federal Parliamentary Inquiries into betting have been conducted in Australia, each of which has attracted significant media attention. The first was *The Joint Select Committee Inquiry into the Advertising and Promotion of Gambling Services in Sport* concluding in 2013. This inquiry aimed to address the amount of sports betting advertising, the exposure of children (and young men) to sports betting advertising, the integration of sports betting advertising into sports commentary, and the effect the saturation and integration of sports betting has on the integrity of, and attitudes towards, sport [32]. Nine recommendations resulted from the inquiry, including commissioning research to examine the long-term effects of gambling advertising on children, with a particular emphasis on the normalisation of gambling, the need for a review of the current self-regulatory action of the sports betting industry with a view to legislation, and a revision of current regulatory exemptions for gambling advertising within sports programs [32].

The second inquiry related to illegal and offshore betting. *The Review of Illegal Offshore Wagering* which concluded in 2015, investigated the economic impact of offshore sports betting on Australian sports betting businesses, the potential regulatory and legislative changes that could be applied in an Australian context, and approaches which could mitigate the risk of negative consequences to consumers [30]. As a result of the inquiry, the Australian Government supported recommendations relating to the development of a National Consumer Protection Framework (containing such measures as a national self-exclusion register for online sports betting, a pre-commitment scheme for online sports betting and a prohibition on lines of credit being offered by sports betting companies); amending laws to emphasise the illegality of offering gambling products to australians, and introducing measures to minimise illegal offshore sports betting [33]. The inquiry was, however, heavily criticised for failing to take into account the proliferation and impact of gambling advertising and marketing [34].

These two inquiries were accompanied by heightened debate and discussion in the Australian national media about the growth of sports betting. The media attention surrounding these two Australian inquiries provides two important case studies from a public health advocacy perspective, as they allow the examination of how gambling is reported by the media, any similarities and differences in perspectives from various stakeholders, and whether there might be areas where public health advocates could more effectively respond to these perspectives.

### Media advocacy: an important component of public health advocacy

Advocacy plays a significant role in public health and, in part, seeks to facilitate change in ‘upstream’ factors such as public policy and practice [11]. One component, media-based advocacy, is often recognised as fundamental to the success of public health advocacy campaigns [35–38] and is used to “...strategically apply pressure for policy change” [38, p. 293]. Media advocacy is important as it can change the perspective of an audience (readers or viewers), enhance support for public health strategies, and counter the views of industries or stakeholders that may oppose reform [39]. For example, research indicates that news reporting of public health issues may both directly and indirectly shape notions of importance in relation to particular issues, and gives stakeholders a space to present their perspectives [40].

Further research has highlighted how groups that contribute to discussions appearing in the media often have competing views, and ultimately influence the information to which audiences have access, and in instances where there is a lack of alternative viewpoints or a consistent message is present, the dominant argument is much less likely to be rejected [41]. Further, research in other areas of public health has demonstrated the influential role of media-based advocacy in influencing public policy and community opinion [42–44]. Research highlights how engagement in media-based advocacy can have a positive impact on policy implementation [44–46]. However, research also demonstrates that media-based advocacy only remains effective if advocates understand the views of opposing stakeholders, the messaging strategies these stakeholders use to promote key messages, and the most effective ways of countering these messages [40, 42]. This includes how messaging strategies change over time either in support of or opposition to different issues associated with reform. While harmful gambling is recognised as requiring a comprehensive public health response [3, 7, 10], there has been limited strategic focus on how public health advocates can work together to develop clear media-based advocacy strategies.

Only one Australian study has explored how different stakeholders frame issues relating to problem gambling within the media, and the dominance of particular stakeholder voices associated with key themes. This study, by Miller and colleagues [2014] found that solutions proposed by governments and the gambling industry for problem gambling focused predominantly on ‘personal responsibility’ frameworks, and argued that more consistent messaging was needed by public health advocates in order to respond to dominant government and industry discourses [47]. The present study contributes to this gap in knowledge by exploring stakeholder voices, themes and messaging strategies relating to the reporting of the two above Parliamentary Inquiries on the advertising and promotion of gambling during sport, and illegal and offshore wagering.

## Research questions

This study examined five key research questions:

1. What are the key themes in print media reporting of sports betting in Australia?
2. Who are the key stakeholders quoted in relation to sports betting? Are some stakeholder groups quoted more than others?
3. How are issues relating to regulatory reform reported? Are some perspectives or positions reported more often?
4. Is there evidence that different stakeholder groups are supportive of certain positions in relation to regulatory reform of the sports betting industry, its products, and promotions?
5. What can we learn from these findings to shape future public health media advocacy strategies?

## Methods

### Approach

A quantitative content analysis was used to explore the themes, voices and perspectives present within newspaper reports published in 12 Australian newspapers during the two time periods of interest.

### Study sample

#### Inclusion criteria and search strategy

The 12 highest circulating daily Australian newspapers were chosen for inclusion based on the circulation data collated by Roy Morgan Research [48]. These were The Sydney Morning Herald, The Herald Sun, The Australian, The Age, The Daily Telegraph, The Advertiser, The Australian Financial Review, The Courier-Mail, Canberra Times, The Mercury, The West Australian and the Northern Territory News. Articles were identified using the Factiva Database with the following search terms: ‘sports betting’, ‘sports wagering’, ‘regulation’, ‘reform’, ‘advertising’, ‘marketing’, ‘sport’ and ‘inquiry’, for the period 1 December 2012–30 June 2013 (Time Period One) and 1 September 2015–31 May 2016 (Time Period Two). The initial search strategy also included the term ‘gambling’. However due to the volume of responses and the lack of relevance of many of the articles retrieved, we narrowed this search term to focus exclusively on sports betting terminology.

The chosen time periods represented approximately one month prior to and during the inquiries, and one month following the release of the respective inquiry reports. News articles, features, sport and insight articles, opinion pieces and editorials, as well as news review and business articles were included in the search. In instances where duplicate articles were identified, the article from the highest circulating newspaper was included. In instances where identical newspaper articles from the same publisher appeared in different publications, the article from the higher circulating paper was included. We also excluded letters to the editor, newspaper comment sections, racing guides, and advertorials as well as articles that solely discussed other forms of gambling such as EGMs (as the primary aim of this study was to examine how sports betting was reported in the media). Initially, a total of 4184 articles were identified. The finalised search strategy that did not include the term ‘gambling’ returned 647 articles, with 406 articles excluded based on the above criteria, leaving 241 articles which were included in the analysis.

#### Development of the coding framework

A coding framework was developed, based on the model employed by Durrant and colleagues [2003] to map tobacco coverage in Australian newspapers [49]. To develop the coding framework we first reviewed the published tobacco, obesity and gambling literature to identify key components of media-based content analysis. From this we identified four important content categories: (1) general descriptors; (2) overall article theme; (3) who was quoted or mentioned in an article (voices); and (4) overall article perspective relating to reform. We then used an inductive approach (opening coding) [50] to determine which categories were applicable to sports betting. This involved reading the articles and noting the main topics of discussion, key stakeholder voices within the articles, and perspectives in relation to the key themes of the inquiries. This led to the identification of eight thematic coding categories and sub-categories that are presented in Table 1. Using this inductive approach, we identified and subsequently included nine stakeholder voices: (1) the sports betting industry; (2) government officials/politicians; (3) sporting codes; (4) broadcasters; (5) non-government organisations/public health experts; (6) journalists; (7) academics; (8) public figures who had experienced gambling harm (such as former sportspeople); and, (9) individuals who had experienced gambling harm.

**Table 1:** Thematic coding categories and definitions

Category / Sub Category	Definition
Category One – Miscellaneous	
*Background/other:*	General information about the sports betting industry, multiple topics may be discussed, with no one topic being the overall focus.
Category Two - Industry Finance and Partnerships	
*Merging/company partnerships:*	The development or changing nature of partnerships between sports betting companies and other companies or individuals e.g. sports betting company’s partnering with broadcasters.
*Company finances:*	Company finances or changes in financial status.
Category Three – Technology	
*Product development:*	Sports betting product development or advances in technology e.g. development of ‘in-play’ betting applications.
Category Four - Marketing and Communication	
*Normalisation:*	The causes and consequences of the normalisation of sports betting.
*Marketing and promotions:*	The marketing and promotions of sports betting, including advertising, inducements and sponsorship.
*Young men:*	The impact or potential impact of sports betting marketing on young men.
*Children:*	The impact or potential impact of sports betting marketing on children.
*Sanctions for inappropriate marketing:*	The use of fines or sanctions for inappropriate marketing or promotions by sports betting companies (or broadcasters where relevant).
Category Five - Gambling Integrity	
*Illegal offshore betting:*	Illegal offshore betting and the impact this has on legal sports betting.
*Criminal activity:*	Criminal or illegal activity outcomes associated with sports betting.
Category Six - Sports Integrity	
*Player betting/match fixing:*	Match fixing, and the potential impact on the integrity of sport.
*Regulatory reform:*	Proposed regulations to protect the integrity of sport.
Category Seven – Reform	
*Implemented reform:*	Reform or regulatory changes (including industry self-regulation) that have already been implemented.
*Need for regulation change:*	The need for regulation/reform change.
*Parliamentary Inquiry:*	The implications of the inquiries into sports betting, including terms of reference, and key findings and outcomes of the inquiry
Category Eight - Gambling Harm	
*Problem gambling:*	Problems gambling or instances of problem gambling directly relating to sports betting. Includes personal examples or stories highlighting the problem in the community.

Five article perspectives were identified in relation to sports betting regulation and reform: (1) articles with positive framing towards reform (including, a. articles positive about reform which would protect vulnerable sub-populations from gambling harm, b. articles positive about regulatory reform which would protect the integrity of sport, and c. articles positive about regulatory reforms but with no specific reason given); (2) articles that did not state or give an opinion about sports betting regulatory reform regulation; (3) articles that contained mixed views about sports betting regulation; (4) articles that argued for the liberalisation of sports betting regulation; and, (5) articles arguing that the status quo should remain.

#### Application of the coding framework

Author One applied the coding framework across the articles, with 10% of articles randomly coded by Author Two and Author Three. Given expert academic commentary from Author Two in a number of news articles during the analysis period, articles that included any reference to Author Two were not coded by Author Two during this process. Each newspaper article was coded for general article descriptors, theme, voice and perspective. The coding allowed for multiple voices. Where there was the appearance of more than one voice or perspective from the same category a code of ‘1’ was used, and where there were no voices or perspectives from a category a code of ‘0’ was used. Each article was also coded for one primary and one secondary theme. These were crosschecked across the coders. Where there were differences, the coders discussed these until a mutually agreed interpretation was reached.

### Data analysis

Basic statistical analysis was undertaken to determine frequencies of article types and overall primary and secondary themes, perspectives given and voices present. Frequency counts also identified which groups supported each type of perspective. Finally, differences in the appearance of themes, voices and perspectives were identified by performing Pearson’s Chi-squared tests of independence on unpaired data from the larger samples. Fisher’s exact tests were conducted when the contingency table expected cell value was less than five. To establish statistical significance we chose a conservative level of significance for α of 0.0017 (α / *k* = 0.05/29 = 0.0017) [51]. The value of *k* was determined by conducting 29 separate statistical tests and applying the Bonferroni adjustment for testing multiple outcomes [51].

## Results

The general characteristics of the newspaper articles are presented in Table 2. Of the 241 articles identified, 114 (47.3%, mean = 16 articles per month) were in Time Period One and 127 (52.7%, mean = 14 articles per month) were in Time Period Two. Across the two time periods approximately one in five articles were published in the broadsheet The Sydney Morning Herald (*n* = 49, 20.3%), followed by the tabloid The Herald Sun (*n* = 39, 16.2%). The fewest articles were published in the Northern Territory News (n = 4, 1.7%). News articles were the most frequent article type (*n* = 93, 38.6%), while opinion or editorial pieces appeared less frequently (*n* = 11, 4.6%).

**Table 2:** General article descriptors

Newspaper Publication	Time Period One + Time Period Two *N* = 241 (%)^a^	Time Period One: Dec 1 2012–30 June 2013 *N* = 114 (%)^a^	Time Period Two: Sept 1 2015–31 May 2016 *N* = 127 (%)^a^
The Sydney Morning Herald	49 (20)	30 (26)	19 (15)
The Herald Sun	39 (16)	13 (11)	26 (21)
The Australian	38 (16)	6 (5)	32 (25)
The Age	32 (13)	15 (13)	17 (13)
The Daily Telegraph	17 (7)	12 (11)	5 (4)
The Advertiser	16 (7)	9 (8)	7 (6)
The Australian Financial Review	15 (6)	6 (5)	9 (7)
The Courier-Mail	9 (4)	6 (5)	3 (3)
Canberra Times	9 (4)	8 (7)	1 (1)
The Mercury	7 (3)	4 (4)	3 (2)
The West Australian	6 (3)	4 (4)	2 (2)
Northern Territory News	4 (2)	1 (1)	3 (2)
Article Type			
News	93 (39)	50 (44)	43 (34)
Miscellaneous	53 (22)	26 (23)	27 (21)
Sport	46 (19)	25 (22)	21 (17)
Business/ Finance	38 (16)	8 (7)	30 (24)
Opinion/ Editorial	11 (5)	5 (4)	6 (5)

^a^Percentages rounded to the nearest whole number

### Primary and secondary themes within articles

Primary and secondary themes across the two time periods are presented in Table 3. Overall, marketing and communication (*n* = 165, 68.5%) and regulatory reform (*n* = 140, 58.1%) were the main themes present, while articles discussing gambling harm (*n* = 9, 3.7%) appeared least frequently. There was a significant decrease in articles featuring a primary message relating to gambling reform between Time Period One (*n* = 56, 49.1%) and Time Period Two (*n* = 32, 25.2%) [*X* ^2^ (1) = 14.835, *p* < 0.0001]. These articles predominantly referred to issues and concerns relating to the sports betting industry, including match-fixing, or addressing the saturation of advertisements during sporting matches by implementing tighter restrictions on marketing. There was a significant increase in articles with a primary theme of technology across the two time periods. While there was no mention of technology as a primary theme in Time Period One, approximately one in ten articles (*n* = 12, 9.4%) had a primary theme of technology in Time Period Two [*X* ^2^ (1) = 11.336, *p* = 0.001]. These articles focused on product development and technological advances, such as the development of in-play sports betting apps (allowing customers to bet during games via mobile technology) by a range of sports betting companies. There were significant decreases in secondary themes relating to marketing and communications between Time Period One (*n* = 64, 56.1%) and Time Period Two (*n* = 31, 24.4%) [*X* ^2^ (1) = 25.329, *p* < 0.0001].

**Table 3:** Primary and secondary themes relating to sports betting in major Australian newspapers

Theme	Time Period One + Time Period Two Totals *N* = 241 (%)^a^	Time Period One: Dec 1 2012–30 June 2013 N = 114 (%)^a^	Time Period Two: Sept 1 2015–31 May 2016 N = 127 (%)^a^	*p-value*
*Primary Theme*				
Regulatory Reform	88 (37)	56 (49)	32 (25)	**<0.0001** ^**^
Marketing and Communication	70 (29)	33 (29)	37 (29)	0.975
Sport Integrity	25 (10)	11 (10)	14 (11)	0.727
Gambling Integrity	24 (10)	9 (8)	15 (12)	0.311
Industry Finance and Partnerships	13 (5)	3 (3)	10 (8)	0.72
Technology	12 (5)	0 (0)	12 (9)	0.001**
Gambling Harm	5 (2)	2 (2)	3 (2)	1.000^b^
Miscellaneous	4 (2)	0 (0)	4 (3)	0.124^b^
*Secondary Theme*				
Marketing and Communication	95 (39)	64 (56)	31 (24)	**<0.0001** ^**^
Regulatory Reform	52 (22)	18 (16)	34 (27)	0.039
Gambling Integrity	35 (15)	12 (11)	23 (18)	0.095
Sports Integrity	25 (10)	17 (15)	8 (6)	0.029
Industry Finance and Partnerships	14 (6)	2 (2)	12 (9)	0.011
Technology	10 (4)	0 (0)	10 (8)	0.002^b^
Miscellaneous	6 (3)	1 (0.9)	5 (4)	0.217^b^
Gambling Harm	4 (2)	0 (0)	4 (3)	0.124^b^

**Statistically significant level < 0.0017

^a^Percentages rounded to the nearest whole number

^b^Fisher’s exact test

#### Key stakeholder voices

Key stakeholder voices present in media reporting across the two time periods are presented in Table 4. Overall, representatives from the sports betting industry (*n* = 149, 61.8%), and government officials or politicians (*n* = 140, 58.1%) were the voices most frequently represented. Voices from public figures who had experienced harm (such as ex-sportspeople) appeared in six articles (2.5%), and voices from the general population who experienced harm from sports betting appeared in five articles (2.1%). There were significant changes in the representation of stakeholders over time, with an increase in industry voices between Time Period One (*n* = 55, 48.2%) and Time Period Two (*n* = 94, 74.0%) [*X* ^2^ (1) = 16.904, *p* < 0.0001], and also significant increases in voices from sporting code representatives (*n* = 36, 31.6% and *n* = 69, 54.3% respectively) [*X* ^2^ (1) = 12.648, *p* < 0.0001]. Finally, there was also a significant increase in voices from non-government organisations or public health experts between Time Period One (*n* = 7, 6.1%) and Time Period Two (*n* = 27, 21.3%) [*X* ^2^ (1) = 11.333, *p* = 0.001].

**Table 4:** Stakeholder voices present in media articles about sports betting in major Australian newspapers

Category	Time Period One + Time Period Two Totals N = 241 (%)^a^	Time Period One: Dec 1 2012–30 June 2013 *N* = 114 (%)^a^	Time Period Two: Sept 1 2015–31 May 2016 N = 127 (%)^a^	*p-value*
Sports betting industry	149 (62)	55 (48)	94 (74)	**<0.0001** ^**^
Government/ politicians	140 (58)	71 (62)	69 (54)	0.212
Sporting codes	105 (44)	36 (32)	69 (54)	**<0.0001** ^**^
Broadcasters	38 (16)	14 (12)	24 (19)	0.159
NGO/ public health experts	34 (14)	7 (6)	27 (21)	**0.001** ^**^
Journalists	24 (10)	17 (15)	7 (5)	0.015
Academics	21 (9)	8 (7)	13 (10)	0.376
Public figures who had experienced harms from gambling	6 (3)	6 (5)	0 (0)	0.010^b^
Individuals who had experienced harms from gambling	5 (2)	1 (0.9)	4 (3)	0.373^b^

**Statistically significant level < 0.0017

^a^Percentages rounded to the nearest whole number

^b^Result of Fisher’s exact test

#### Article perspectives on reform

The key perspectives featured in the articles and changes over time are reported in Table 5. Almost half (*n* = 116, 48.1%) of articles presented the need for regulatory reform as the main perspective, with 57 (23.7%) of these focusing on the need for regulatory reform of the sports betting industry and its products and promotions to protect vulnerable sub-populations, in particular young men and children. A further 38 (15.8%) articles presented the view that regulatory reform of the sports betting industry was needed to improve, ensure, or protect integrity issues in sport. These included mechanisms to address illegal offshore sports betting or match fixing. The remaining 22 (9.2%) articles supported reform, but did not explain why reform was necessary. In just over one-third of articles (*n* = 81, 33.6%) no preference was stated for either reform or no reform, while only a small number (*n* = 4, 1.7%) stated that current regulations should remain.

**Table 5:** Reform perspectives given in the stakeholder discussions of sports betting in major Australian newspapers

Category	Time Period One + Time Period Two Totals *N* = 241 (%)^a^	Time Period One: Dec 1 2012–30 June 2013 N = 114 (%)^a^	Time Period Two: Sept 1 2015–31 May 2016 N = 127 (%)^a^	*p-value*
Positive for regulatory reform of the sports betting industry, its products or promotions	117 (49)	72 (63)	45 (35)	**<0.0001** ^**^
(a) Regulatory reform to protect vulnerable sub-populations	57 (24)	41 (36)	16 (13)	**<0.0001** ^**^
(b) Regulatory reform to protect the integrity of sport	38 (16)	19 (17)	19 (15)	0.717
(c) Regulatory reform – no specific reason given	22 (9)	12 (11)	10 (8)	0.475
No perspective relating to regulatory reform	81 (34)	25 (22)	56 (44)	**<0.0001** ^**^
Mixed views regarding regulatory reform	28 (12)	7 (6)	21 (17)	0.012
Regulatory liberalisation of sports betting	11 (5)	7 (6)	4 (3)	0.267
Endorsed status quo regarding current regulations	4 (2)	3 (3)	1 (0.8)	0.347^b^

**Statistically significant level < 0.0017

^a^Percentages rounded to the nearest whole number

^b^Result of Fisher’s exact test

There were three instances of statistically significant change appearing across the two time periods. First, significantly fewer articles presented a perspective that regulatory reform was necessary between Time Period One (*n* = 72, 63.2%) and Time Period Two (*n* = 45, 35.4%) [*X* ^2^ (1) = 18.487, *p* < 0.0001], in particular to protect vulnerable sub-populations (e.g. young men, children) from developing problems with gambling or being exposed to gambling marketing at Time Period One (*n* = 41, 36.0%) and Time Period Two (*n* = 16, 12.6%) [*X* ^2^ (1) = 18.164, *p* < 0.0001]. Third, there was a significant increase in articles that did not present any perspective relating to gambling regulation or reform across the two time periods, Time Period One (*n* = 25, 21.9%), Time Period Two (*n* = 56, 44.1%) [*X* ^2^ (1) = 13.226, *p* < 0.0001].

Finally we explored which stakeholders held different perspectives in relation to gambling reform (Table 6). Key proponents of tightening gambling regulation to protect the Australian community included politicians, academics, non-government groups and in some instances individuals who had experienced gambling harm. A number of politicians from a range of perspectives, including left, conservative and independents focused on the impact that offshore illegal sports betting had on gambling revenue in Australia. They also supported calls for regulations to address integrity in sport issues. Similarly, sporting codes, broadcasters, individual journalists and public figures who have experienced harms from gambling supported these reforms. Sporting codes and the sports betting industry argued that no tightening of gambling regulation were necessary, due to their implementation of self-regulation measures.

**Table 6:** Stakeholder views about Australian sports betting regulation

Stakeholder Group	Regulatory reform to protect vulnerable populations	Regulatory reform to uphold integrity of sport	Reform not specified	Not expressed	Mixed views	Liberalise regulation	No change
*State Government*	✓			✓	✓		
*Federal Government*		✓	✓		✓		
*Greens Party*	✓						
*Government – Not specified*						✓	
*Greens Party Members of Parliament*				✓			
*Independent Members of Parliament*	✓		✓				
*Federal Government Organisations*				✓			
*Independent Senators*	✓	✓					
*Greens Party Senators*	✓	✓	✓	✓			
*Experienced Harm - General Population*	✓						
*Experienced Harm - Public Figures*	✓	✓					
*Journalists*	✓	✓	✓	✓	✓	✓	
*Academics*	✓		✓		✓		
*Non - Government Organisations*	✓			✓	✓		
*Peak Bodies*	✓				✓		
*Federal Police*		✓					
*Broadcasters*		✓			✓	✓	
*Sports Betting Industry*				✓	✓		✓
*Sporting Codes*		✓	✓	✓	✓	✓	✓

## Discussion

This study sought to explore how sports betting is reported in major Australian newspapers, with a view to informing future public health advocacy approaches. Although media advocacy is recognised as a key component in successful public health interventions and initiatives [35, 36], there has been limited discussion about how public health academics and professionals can more effectively utilise media-based advocacy strategies to respond to government policies and industry tactics in the area of gambling. Findings from this study raise a number of key points for discussion.

The first relates to sports betting-related news articles. Data analysis revealed that the Northern Territory News published four articles relating to sports betting during the collection period, representing the smallest number of articles overall, three of which occurred during Time Period Two. Given the proportion of sports betting companies registered in the Northern Territory [16], it is interesting that there appears to be limited discussion in the local media in relation to this industry. Further research should investigate how different media outlets in different geographical regions (both nationally and internationally) frame debates relating to gambling. This could also include how media outlets with more gambling advertising report issues associated with gambling as compared to those with less gambling advertising.

The second point relates to the key themes identified. Findings suggest that even when major government inquiries focus on issues other than the marketing and promotional tactics used by the sports betting industry, this issue remains high on the media agenda. Research indicates that the media report on issues they believe are both newsworthy and of community interest, thus influencing how an issue is perceived by the public [52]. Given that reporting about sports betting marketing and promotional tactics remained a continued point of interest, even during the *Review of Illegal Offshore Wagering* [30], this suggests that sports betting marketing and promotions are still considered by the media to be an important issue of public concern. Regulatory reform regarding the marketing of sports betting during sporting matches has been identified as a key priority for public health academics [18, 32], politicians [21, 53], and community organisations [32]. Recent studies have continued to highlight the impact of marketing on the sports betting behaviours of young men [6, 14] and young people [5, 18]. Given (1) the role that media-based advocacy plays in shaping and influencing public policy decisions and challenging public perceptions, and (2) the continued media interest regarding the impact of gambling industry marketing tactics, public health researchers should seek to work more closely with media outlets to ensure that evidenced-based findings from research relating to marketing and promotions are featured in media reports.

There were few news articles specifically focusing on issues relating to gambling harm across both time periods. This may be due to the effectiveness of industry and associated stakeholder voices in shifting the focus to other issues such as integrity. This is a known tactic of industries such as tobacco, which have historically attempted to shift the focus from harmful products and downplay any associated harm [54, 55]. However it may also be because public health and social organisations, researchers and advocates have failed to provide the media with adequate information about the consequences of harmful gambling. Providing media training to those who have experienced gambling harm may contribute to increasing the focus on harmful aspects of gambling for individuals, communities, and populations. This has been a successful strategy in other public health advocacy initiatives [46, 56, 57]. The increase in themes relating to technology between Time Period One and Time Period Two, is perhaps unsurprising given the nature of the *Review of Illegal Offshore Wagering* (during Time Period Two) [30] in which concerns were raised about the availability and growth of sports betting-related technologies. However, of note was a significant decrease in articles with a primary theme of regulatory reform between Time Period One and Time Period Two. Public health advocates should consider how to more effectively maintain a media presence around issues of significance.

The third discussion point relates to key stakeholder voices presented in the media. The sports betting industry was the stakeholder ‘voice’ identified most frequently. This is perhaps not surprising given that media reporting typically seeks comment from industry at the centre of policy or regulatory change. However, by Time Period Two, there were also significant numbers of voices from sporting codes who, for the most part, held similar perspectives to the sports betting industry regarding the liberalisation of gambling regulations. Given identified links between the sports betting industry and sporting codes [18], the increase in voices from various sporting codes is perhaps unsurprising due to the financially beneficial relationships between sporting codes and sports betting companies [58]. This poses a challenge for public health advocates, with research indicating that those stakeholders who have the loudest voice and that garner the most attention also have the greatest potential to influence decision makers [59]. There is still a significant disparity between the number of times representatives from the sports betting industry and sporting organisations are quoted, as compared to those who argue that increased reform is needed to protect and prevent gambling harm. Given that there are still very few public health experts working in the area of sports betting, those advocating for reform should work together in presenting unified messages relating to regulatory reform and providing a comprehensive, evidenced-based responses to industry messages. Lessons from tobacco and other areas include the need for advocates to ‘sing from the same song sheet’, provide clear robust evidence, and comprehensively respond to industry messages [60–62].

There is also a role for coalitions to support community stakeholders in providing comment for media reporting. Community voices are critical in bringing human stories to public health advocacy initiatives [56, 57]. Some studies examining media advocacy attempts in the alcohol industry identified that initiatives are most effective when ‘authentic voices’ are telling ‘real local stories’ that the public can connect with [46]. Holder and Treno [1997] argue that such news stories provide a more salient and credible message than news stories about events with which people cannot identify [46]. Other studies have reported that the magnification of community voices enables pressure to be placed on key decision makers that in turn encourages policy change [63]. However, we found very little evidence of voices from those who had experienced gambling harm represented in media reporting of sports betting. Where these did appear they were predominantly high profile individuals such as former professional sportspeople. The stigma around problem gambling may be a contributing factor to the limited number of individuals that publicly share their own experiences of harmful gambling. Public health advocates could also seek to engage a much broader range of voices and perspectives from the community. For example, research shows that parents (and children) are concerned about the impact of sports betting marketing on young people [5]. Advocates should consider how these groups could be supported to share their concerns with journalists.

The final point for discussion relates to perspectives taken within the media. In Time Period Two there was a significant decrease in articles that presented a perspective that sports betting reform was necessary to protect vulnerable population sub-groups. This shift in perspectives may be attributed to the increase in voices from the gambling industry and sporting codes – which in part relate to the terms of references of the *Review of Illegal Offshore Wagering* [30]. Our study shows that many stakeholders such as independent politicians and members of non-government organisations continue to advocate for regulatory reform. Public health advocates should consider how they could more effectively work with these stakeholders to create clear and cohesive media messaging strategies to counter those of the sports betting industry. They should also consider organisational and other mechanisms to ensure that strong, evidence-based coalitions continue to present consistent messages both over time and when specific opportunities for reform arise.

Some study limitations should be acknowledged. The use of Factiva as a tool for the collation of articles for analysis was restrictive, as it does not allow for consideration of how an article’s placement, size and attached imagery impacts how the article is perceived. Another limitation relates to the two discrete time periods in which data was collected. As a consequence of the specific time periods, the articles are not representative of discussions about sports betting more broadly, thus limiting the generalisability of this study. In addition, this study also excluded letters to the editor and newspaper comments sections. In doing so, there was potential for the perspectives of the community to also be excluded. Finally, our study was only conducted on newspapers that occurred in printed form and did not include newspapers accessible via online sources only. As a result, articles published by freelance independent journalists may not have been included in the analysis.

## Conclusion

Findings from this study indicate that, to some extent, various key stakeholder groups such as state governments, sporting codes and individuals who have experienced harm from sports betting agree that reform is necessary. However, there is a lack of consensus in relation to what type of reform is required and who in particular supports it. By exploring this, public health advocates will have the opportunity to effectively mould their advocacy approaches, thus increasing the prospects for successful policy change and regulation. Public health advocates should consider how appropriate messaging through the inclusion of personal (and relevant) stories will improve future media advocacy, and in responding to messages from the gambling industry. Finally, it will be important to ensure consistent, evidence-based approaches over time to working with the media.

## Abbreviations

EGMs: Electronic Gambling Machines
PHAA: Public Health Association of Australia
VRGF: Victorian Responsible Gambling Foundation
